# Protected area networks do not represent unseen biodiversity

**DOI:** 10.1038/s41598-021-91651-z

**Published:** 2021-06-10

**Authors:** Ángel Delso, Javier Fajardo, Jesús Muñoz

**Affiliations:** 1 Universidad Internacional Menéndez Pelayo, Madrid, Spain; 2 Real Jardín Botánico (RJB-CSIC), Plaza de Murillo 2, 28014 Madrid, Spain; 3 UN Environment Programme World Conservation Monitoring Centre (UNEP-WCMC), Cambridge, UK

**Keywords:** Biodiversity, Conservation biology, Ecological modelling

## Abstract

Most existing protected area networks are biased to protect charismatic species or landscapes. We hypothesized that conservation networks designed to include unseen biodiversity—species rich groups that consist of inconspicuous taxa, or groups affected by knowledge gaps—are more efficient than networks that ignore these groups. To test this hypothesis, we generated species distribution models for 3006 arthropod species to determine which were represented in three networks of different sizes and biogeographic origin. We assessed the efficiency of each network using spatial prioritization to measure its *completeness*, the increment needed to achieve conservation targets, and its *specificity*, the extent to which proposed priority areas to maximize unseen biodiversity overlap with existing networks. We found that the representativeness of unseen biodiversity in the studied protected areas, or *extrinsic representativeness*, is low, with ~ 40% of the analyzed unseen biodiversity species being unprotected. We also found that existing networks should be expanded ~ 26% to 46% of their current area to complete targets, and that existing networks do not efficiently conserve the unseen biodiversity given their low specificity (as low as 8.8%) unseen biodiversity. We conclude that information on unseen biodiversity must be included in systematic conservation planning approaches to design more efficient and ecologically representative protected areas.

## Introduction

The biodiversity crisis is now beyond discussion^1,2^. Around one million of all described species (representing 25% of all assessed species) are estimated to be threatened^3^, a striking figure that emphasizes the need for increased conservation efforts. One of the most successful approaches to slow or stop species loss and ecosystem degradation is the establishment of protected areas (PAs)^4^. In recognition of this, nations worldwide agreed to a target to protect at least 17% of all lands and 10% of seascapes by 2020 through ecologically representative systems of PAs^5^. By 2018, 14.9% of global terrestrial lands were included in PA networks, nearing the target set for 2020^6^. Despite this, there is mounting concern that satisfactory biodiversity outcomes will not be achieved not only in countries and regions that have not met their target, but also in the many that have^6–8^. This situation stresses the importance of networks being ecologically representative, and illustrates the complex endeavor of designating new PAs, which includes reconciling opposing views and managing shortages of funds and time. In the post-2020 biodiversity framework^9^, this concern has raised the argument that new targets should focus on increasing the effectiveness of biodiversity conservation, emphasizing the value of lands selected for preservation^10^ to promote quality areas that enhance network properties such as representation.

Since the amount of area that can be protected is limited, it is crucial that the sites selected for conservation are efficient and representative of the three elements of biodiversity: genetic, species, and ecosystem diversity^11–13^. However, most existing PAs established before the 1980s were designed following criteria that aimed to protect “wild areas”^14^ or that were based on aesthetics, economics or socio-politics rather than representation^11^. As a result, the global PA network is biased towards certain biomes and ecoregions^4^, with an overrepresentation of high elevation or remote places where conflict with alternative land uses is low^15^. The bias of PA networks has raised doubts on their general effectiveness with regard to conservation of biodiversity representativeness (e.g.,^16,17^). More recently, however, the scope and design of PA networks in the last few decades have shifted towards more scientifically sound objectives, including representativeness^4^. A major milestone in the incorporation of biodiversity representativeness in conservation planning is the mainstreaming and formalization of the principles of Systematic Conservation Planning (SCP)^12^, a framework that promotes efficient conservation decision making that is based on explicitly declared scientific targets.

When representativeness has been an explicit criterion for PA designation, it has been usually applied to the most well studied or charismatic taxonomic groups, such as mammals, birds or vascular plants, or coarse filter biodiversity surrogates such as ecosystems^11,18,19^. As a result, PA network representativeness is biased towards taxa that are usually among the least diverse^19^, and worse, that are poor surrogates for representativeness of highly diverse but less showy or less studied taxa, such as arthropods, mollusks and annelids^18,20–22^. The aforementioned groups, among many others, account for most of the “unseen” or “hidden” biodiversity, and are rarely, if at all, included in conservation decisions^23,24^, breaching the criterion of representativeness. This problem is compounded if we consider that extinction rates are higher among the groups that contribute more to the unseen biodiversity^25–28^. Nonetheless, these taxa are important in conservation planning because they represent the majority of the biodiversity in any given area, inhabit all types of environments, and fulfil essential functions for the maintenance of many ecosystem services of immense economic value for humans. Indeed, the annual economic value of pollination, dung burial, pest control, and recreation services provided by insects alone in the USA has been estimated at $57 billion^29^. Other authors have estimated that, at the national scale, the pollination service provided by insects ranges from 1 to 16% of the market value of agricultural production^30^. Another one of nature’s contributions to people, although challenging to valuate, is the (unseen) biodiversity of saprotrophic fungi and soil invertebrates (e.g., nematodes, mites, collembolans, annelids, myriapods), and unseen biodiversity their intricate interactions, which are crucial for the maintenance of soil fertility^31^.

Unseen biodiversity has been excluded from conservation planning partly due to the knowledge gap for the not-so charismatic groups. Two types of shortfalls affect this gap and hinder the ability of planners to include these groups in conservation planning: first, the Linnean shortfall, as there are still many undescribed species; and two, the Wallacean shortfall, given that knowledge about the distribution of many species is patchy^32^. However, spatial information for unseen biodiversity groups is growing (e.g., insect records added to GBIF per year grew from 1.6 million in 2000 to 5.8 million in 2014; www.gbif.org). Despite some limitations, such raw distribution data can be used in SCP, and are even more valuable when combined with the use of species distribution models (SDMs)^33^. SDMs relate georeferenced observations of well-identified individuals, although limited in number, to relevant ecological predictors, to produce suitability maps that show where a species might be found. This information, combined with data on actual species distribution, can reduce the existent information gap.

Once there is enough information on species distributions, either from actual data points or from data derived from SDMs, it is possible to test the ability of PA networks to represent different elements of biodiversity. This type of analysis typically starts with a representation analysis in which conservation features are classified as either represented or not in a given PA network on the basis of overlapping distributions^12^. Here, we define *intrinsic representativeness* as the degree of representation of the biodiversity elements considered by planners when they first designed a given network. As the notion of conservation planning aimed at representing elements of biodiversity is relatively new, and acknowledging that it has not been considered in the establishment of most existing PAs, we extend the concept of *intrinsic representativeness* to include the elements of biodiversity commonly represented in conservation planning, typically terrestrial vertebrates and charismatic or umbrella species. In fact, the lion’s share of the funds dedicated to biodiversity conservation are still spent on these types of species^34^. The question is whether the so generated PA also maximizes the representativeness of other elements such as unseen biodiversity, which is typically not considered in the design process. To address this, we define *extrinsic representativeness* as the degree of representation of unseen biodiversity groups. Related to this is the question of whether a PA network efficiently represents a group of species such as unseen biodiversity ones. Several SCP methods can be used to identify clusters of priority conservation areas that optimize representativeness for a given group of species (i.e., those that include a maximal percentage of the species represented, while minimizing costs). These methods can also be used to estimate specific shortfalls of current PA networks by exploring which areas should be added to the network, or to identify group-specific optimal representation areas, which can be contrasted against actual PAs to assess their specificity by determining the degree to which they overlap.

The objective of this study is to assess the potential of existing PA networks to correctly represent the unseen biodiversity. Specifically, we test (1) the extrinsic representativeness (i.e. of taxa not used for designation) of existing PA networks, and (2) the overall efficiency of these networks from two perspectives: (i) *representation completeness*, by exploring how much area would need to be added to current PAs to represent unseen biodiversity; and (ii) *representation specificity*, by evaluating the extent to which the most efficient conservation areas (i.e., those identified using a spatial prioritization algorithm) designed using exemplary groups of unseen biodiversity overlap with existing PA networks.

Our hypothesis is that the almost-exclusive use of charismatic groups in SCP does not result in truly representative PA networks, confirming the importance of including unseen biodiversity groups in SCP to increase both intrinsic and extrinsic representativeness of PA networks.

## Results

**Figure 1:**
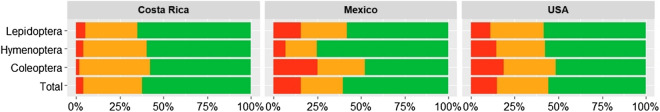
Conservation targets achieved by the three test countries by insect order and country total. Orders from top to bottom: Lepidoptera, Hymenoptera, and Coleoptera. Green: extrinsic representativeness (100% conservation target achieved); orange: underrepresented species (> 50% but < 100% conservation target achieved); red: neglected species (≤ 50% conservation target achieved).

The extrinsic representativeness of existing PAs was ~ 60% for the three test areas, with slight differences observed among countries (Fig. 1). Average extrinsic representativeness was highest for Costa Rica (62.28%), followed by Mexico (60.47%) and the USA (56%). Average representativeness also varied across taxa by countries: 56% for Hymenoptera, 54% for Lepidoptera, and 42% for Coleoptera. Coleoptera was the worst represented order at all sites, and also the one with the most neglected species (i.e., with less than half of their target met; see “Methods”). The best represented order in the USA and Costa Rica was Lepidoptera, while Hymenoptera was the best represented in Mexico, with more than 75% of the analyzed species satisfactorily included in PAs. Costa Rica had the fewest neglected species overall (< 5%). However, about 25% of the Coleoptera and 17% of the Lepidoptera in Mexico were classified as neglected, as were ~ 20% of the taxa of each of the three orders in the USA.

**Figure 2:**
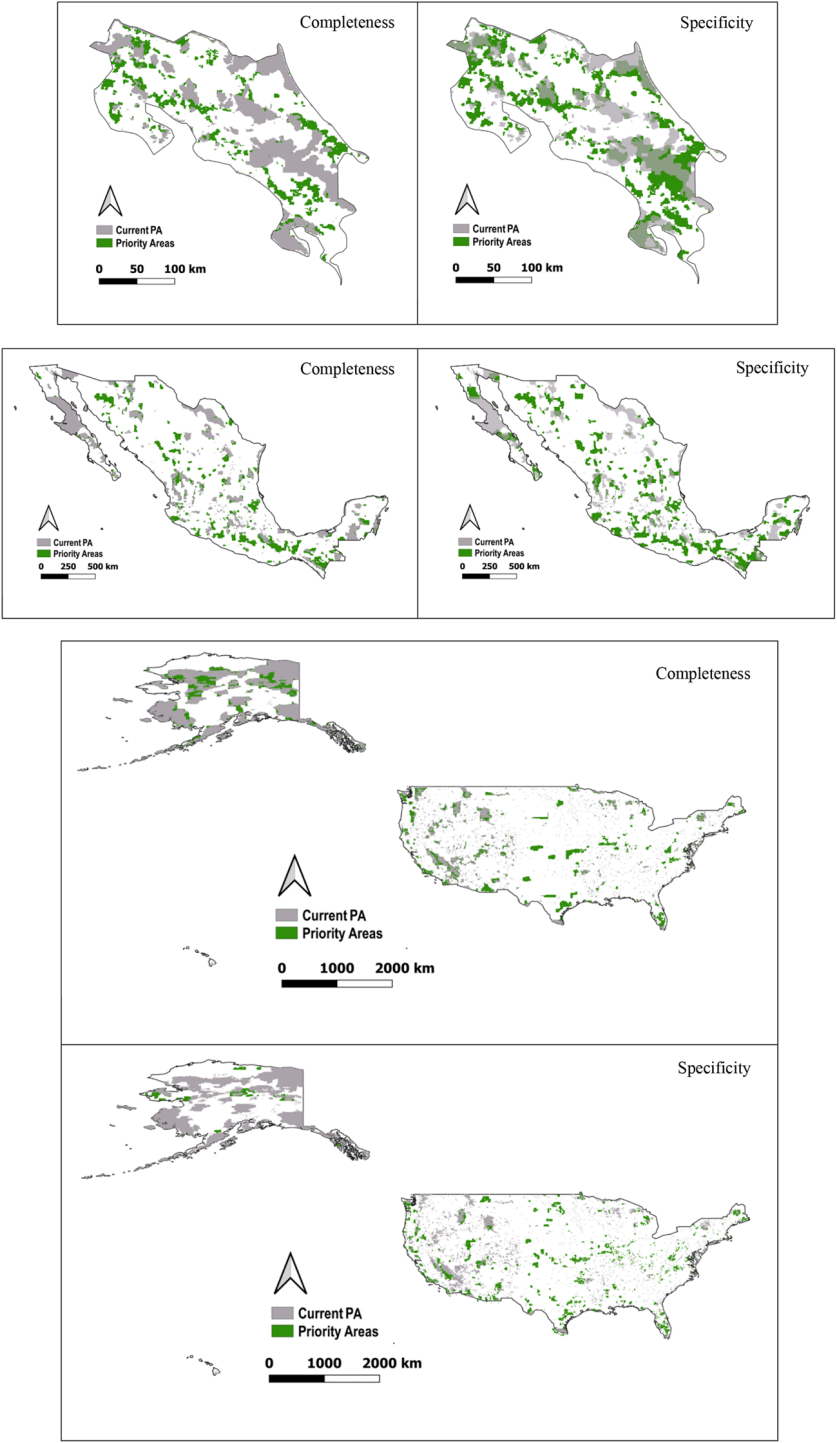
Maps of the test countries showing proposed priority areas for unseen biodiversity (green) and existing PA networks (gray) in terms of completeness and specificity. As shown in the completeness maps, priority conservation areas complement existing PAs to achieve conservation targets. By contrast, the specificity maps show that priority conservation areas, which optimize the conservation of some elements of unseen biodiversity, do not necessarily complement or overlap with existing PAs. Maps created with Quantum GIS v. 3.4.1 (www.qgis.org).

The efficiency of the existing PA networks also differed by country and taxa for the two perspectives evaluated (Table 1). *Representation completeness* was lowest for Mexico, which would require an expansion of 46.34% of its current PA to achieve unseen biodiversity targets. For Costa Rica and the USA, completeness was higher, although both would need to increase their existing PA coverage by around a third (26.28% and 29.82%, respectively) to fully represent unseen biodiversity, if existing PAs are included in the proposed solution (Fig. 2).

**Table 1:** Percentage of extrinsic representativeness and efficiency in terms of representation completeness and specificity estimated for the three test countries.

		Costa Rica	Mexico	USA
Extrinsic representativeness	Unseen biodiversity targets achieved by the existing protected area network (%)	62.28	60.47	56
Efficiency	Completeness (%)	73.72	53.66	70.18
	Specificity (% overlap with existing PA)	49.88	20.1	8.8

*Representation specificity* was highest for Costa Rica, with ~ 50% of the priority areas for unseen biodiversity overlapping with the existing network. This value dropped to 20.1% for Mexico and plummeted to 8.8% for the USA (Table 1).

## Discussion

This study reports the first wide-scale attempt to assess unseen biodiversity representativeness and PA representation efficiency. By assessing three PA networks with varied characteristics, we demonstrated that existing PAs have considerable gaps, in terms of area, to protect highly diverse taxa (which are usually ignored in SCP), and limited efficiency in representing this diversity. Almost half of the analyzed species are not well represented in existing PA networks, and around 15% of them are neglected, demonstrating a deficiency in extrinsic representativeness for unseen biodiversity. To improve extrinsic representativeness, large amounts of area would need to be incorporated into existing PA networks or the network configuration would need to be changed substantially, which is not entirely surprising given that unseen biodiversity has hardly played a role in previous PA planning and designation.

Our finding that about 40% of the analyzed unseen biodiversity species are not represented at all in current PA networks is consistent with the results of other studies that focused on other unseen biodiversity groups. For instance, D'Amen et al.^35^ found that 87% of the studied saproxylic beetles are not represented in the Italian PA network; Martín-Piera^36^ reported that five endemic dung beetle species are underrepresented in Spain; and Kohlmann et al.^23^ showed the presence of conservation gaps for dung beetles in Costa Rica. In terms of funding, a recent study showed that European Union conservation funds are strongly biased towards charismatic species, with birds and mammals alone accounting for 72% of species and 75% of the total budget, and that two of the species receiving the most money (the brown bear and the grey wolf) are not even threatened, according to the IUCN^34^. The lack of adequate representativeness coverage by existing PA networks has also been shown for other taxa, though it is less severe than that found for unseen biodiversity groups. For example, a study of conservation targets in Costa Rica showed that just 25% of the targeted mammalian species are not covered by its PA network, even though the authors used more demanding targets than we did^37^, and only 18% of mammals in Mexico are unprotected by current PAs^38^. This demonstrates that current PA networks, although not explicitly defined on the basis of charismatic taxa (in most cases), are better at protecting these animal groups than the less showy ones. Relying more or less exclusively on well-known and charismatic species introduces an additional bias for poorly known systems for which species data are scarce. This is the case for terrestrial ecosystems in poorly studied regions such as humid tropical forests, but also for “next door” ecosystems that are almost completely composed of unseen biodiversity groups, such as freshwater ones, which are often neglected in conservation plans despite the existence of thorough SCP studies focused on them^39^.

These differences reinforce the idea that conservation gaps are higher for species not included in SCP analyses, and also challenge the capacity of charismatic groups to truly represent unseen biodiversity the distribution and richness patterns of unseen biodiversity species, as shown by Escalante et al.^40^. Although our study does not directly compare representation between seen and unseen biodiversity, our results support the existence of this bias, as suggested by previous research. Spatial distributions of vertebrate and ant diversities do not match in Florida^20^, mosses in Europe show a reversed latitudinal richness pattern compared with vascular plants^41^, freshwater invertebrates in the Amazon of Ecuador peak at higher elevations than vertebrates^42^, and beetle and polypore species richness is poorly related to bird species richness in boreal forests^43^. The mounting evidence emphasizes the need to include unseen biodiversity in SCP approaches in order for PA networks to be truly representative of biodiversity. If distribution data for inconspicuous taxa are not available or are poor, close surrogates should be included, such as better known species of the same family or genus^44^, but not species of taxonomically unrelated groups.

Our methods can be consolidated in a novel framework in which to assess the efficiency of PA networks to represent subsets of biodiversity by focusing on what we have termed here as extrinsic representativeness. The framework is conceived around two complementary spatial analyses involving the identification of optimal areas for the representation of a certain target group of conservation features, which can be obtained with the help of a site-selection algorithm. The first analysis (*completeness*) aims to assess the extent to which a PA network needs to be expanded in order to achieve complete representation of a target group. To do this, we start by identifying optimal areas for expansion of current PAs, then we calculate the magnitude of the expansion required. With this approach, we can identify PA networks that are close to completion (in terms of representation). A combination of factors influences the ability of a PA network to reach completion including the efficiency of the network in representing the target group, those related to biodiversity patterns such as species richness and turnover, and the current configuration of the PAs within the network. For instance, regions with high species richness and beta diversity will potentially need larger expansions. Such regions require large areas of protection because their biodiversity generally does not overlap due to high replacement, reducing the number of truly complementary opportunities^45,46^.

The second spatial analysis (*specificity*) evaluates a network’s efficiency in representing a target group by measuring how well proposed optimal PAs for unseen biodiversity are reflected in the current PA network. The idea is that highly congruent networks are indicative of high specificity in the representation of the target group, whereas a general spatial mismatch indicates poor specificity. This method has been commonly applied to evaluate PA representation efficiency for whole countries or regions^47,48^. However, these analyses typically focus on maximizing the representation of taxonomic groups for which comparably more spatial data is available, such as terrestrial vertebrate species. Here, we propose adapting this analysis to other taxa, particularly those comprising unseen biodiversity, to assess efficiency in capturing extrinsic representativeness. In theory, efficiency in representing groups of species depends largely on how distinctive is the distribution pattern of the evaluated group compared with that of those elements that have been historically more influential in the establishment of PAs. In this context, conducting this analysis on taxonomic groups or conservation features with very unique distributions (e.g. freshwater biodiversity, uncharismatic species, soil biodiversity or agrodiversity, among others) can potentially reveal low specificity levels of extrinsic representation. Lastly, considering *completeness* and *specificity* together, along with a standard representation gap analysis, provides a means to comprehensively evaluate the efficiency of PAs in representing a group of conservation features. For instance, in our results, the gap to completion of the networks in Costa Rica and the USA is similar, as both require an equivalent expansion in percent terms (see Table 1). However, it should not be assumed that the PA network of Costa Rica is less efficient than that of the USA because it needs a large expansion, especially considering its network accounts for a far greater percentage of the total area in comparison (Table 2). Indeed, the specificity analysis indicates that PAs in Costa Rica are more specific in representing unseen biodiversity. Therefore, the proposed framework is able to diagnose different types of shortfalls: the PAs in the USA are inefficient in representing unseen biodiversity because they are largely not specified for this objective and because they fall short in extent. By contrasting, the network in Costa Rica showed the highest specificity; however, due its high level of biodiversity, it still needs a considerable expansion to efficiently represent all of the evaluated species.

**Table 2:** Comparative metrics of the three test areas. Sources for insect species richness: Costa Rica^59^, Mexico^60^, and the USA^61^.

	Costa Rica	Mexico	USA
Current protected area (% of total area)	27.60%	14.16%	12.99%
Area (km^2^)	51,100	1.97 * 10^6^	9.83 * 10^6^
Insect richness (described/estimated)	68,500/365,000	47,800/97,000	91,000/164,000
Number of described insect species/total area	1.341	0.024	0.009

Unseen biodiversity plays important roles in ecosystem maintenance and in services to society, such as nutrient cycling, primary production, soil formation, habitat provision, and pollination. We conclude that unseen biodiversity should be explicitly considered in the designation of PA networks in order to make them more representative but also more functional. Consideration of unseen biodiversity could also help in the design of more efficient restoration strategies. We acknowledge that information is poor or at a coarse scale for many of the groups providing these services (e.g., fine-grained distributions of pollinators), however, given their importance to nature and society, funding programs that close the taxonomic gap are worth the investment^49–53^. Until more data are available, we argue that SDMs, despite some limitations, represent a valuable source of information to overcome data gaps and to inform conservation planning. However, we must highlight a few methodological considerations as these models represent potential distributions of species and model parameters do not fully reflect several factors^54^. The most important factors are: (1) evolutionary (geographic barriers and speciation processes), (2) ecological (biotic interactions), and (3) anthropogenic (habitat degradation). Consequently, these models can result in an overprediction, especially in areas and groups with a low sampling effort^55,56^. We demonstrated, however, that potential problems can be minimized by simulating the effect of geographic barriers^57^ and by excluding sites in which ecosystems have been degraded from priority areas, thereby ensuring that only areas that maintain a certain level of ecological integrity are considered.

In conclusion, this study reveals the varying degree of representativeness and representation efficiency of three insect groups in three countries, highlighting the need to consider unseen biodiversity in conservation planning. Although these groups represent only a small part of the unseen biodiversity, we hypothesize that results found with almost any other taxonomic group comprising unseen biodiversity, and for any geographic area and resolution, will be similar to those found here. Finally, we emphasize the need for increased efforts to describe and sample other taxonomic groups, and for mainstreaming their inclusion in future conservation planning.

## Methods

### Testing areas

Costa Rica, Mexico, and the USA were the test areas used to evaluate the representativeness of the unseen biodiversity in PA networks. These countries met three criteria that allow a richer portfolio of contexts relevant to the research question to be explored: (1) high availability of georeferenced data on megadiverse taxa, something uncommon for most countries, (2) follow a latitudinal gradient from tropical to temperate climates associated with a decrease in species diversity, and (3) PA networks have different sizes (Table 2).

### Unseen biodiversity taxa selection

Selecting taxa of unseen biodiversity for evaluation was difficult. Almost by definition, unseen biodiversity groups are poorly represented in public databases (e.g. GBIF); thus, the challenge was to find highly diverse taxa with enough information on distribution for all three test areas. For some groups (e.g., bryophytes), the information was good for one area but poor for the other(s), and information for most groups was poor worldwide. We explored insects as suitable candidates as they account for 5 to 6 million of the 11 million animals described to date^19^. In addition, they are found in nearly all terrestrial ecosystems worldwide, and they provide fundamental ecosystem services^29^. Although it is estimated that less than 30% of extant insect species have been formally described^58^, certain insect orders are reasonably well known and represented in public databases. Moreover, studies have shown that insect richness at higher taxonomic ranks, such as genera or families, are good surrogates for total insect biodiversity at the species level^44^. Finally, insect biodiversity is reasonably well known for the three test areas, and it follows the latitudinal gradient criteria described above (Table 2). Thus, we concluded that insects represent an appropriate group with which to test extrinsic representativeness coverage in existing PA networks and guide conservation assessments.

We evaluated the representation of 1,002 species for each test area. Species were randomly selected from three of the most diverse orders of insects, Coleoptera, Hymenoptera and Lepidoptera, excluding exotic species listed in national catalogues^62,63^ and the IUCN online database (iucngisd.org). These orders were selected because the largest and most comprehensive sets of georeferenced data are available for these orders compared with other insect groups, and because they are diverse, allowing the greatest heterogeneity of niches and ecoregions to be represented in our analyses. For each order, we downloaded occurrence data from various sources (see below), discarding species with less than 15 unique occurrences at the pixel resolution of the environmental layers^64,65^, a practice common in studies combining SCP and SDMs^66,67^. After discarding these species, 334 were randomly selected for each order, for a total of 1002 species per study area (Supplementary Table 1). As the availability of data on the chosen taxonomic classes and countries was highly heterogeneous, we used a random, constant, and relatively high number of species across test areas to counterbalance any potential impact of biases in our results.

### Species data

We built a database of georeferenced occurrence points, obtained mainly from the Global Biodiversity Information Facility (GBIF), for Costa Rica (https://doi.org/10.15468/dl.zropvg), Mexico (https://doi.org/10.15468/dl.kgaeqh), and the USA (https://doi.org/10.15468/dl.7vadxq). We also included records within a 100-km buffer zone around the borders of each country to avoid artifacts related to political borders in the SDM. The database was complemented with additional occurrence records gathered from diverse sources: Rosser et al.^68^ for species of Heliconiine, the “Datos Abiertos” database (datos.gob.mx) for insects in Mexico, Camero & Lobo^69^ for dung beetles in Costa Rica and Mexico, and the project Biodiversity Information Serving Our Nation (BISON, bison.usgs.gov) for species in the USA. To minimize the number of incorrect records, which are typical in public databases^70^, we used the package CoordinateCleaner^71^ in the R environment (v 3.4)^72^ to remove records located at country centroids, natural history museums or research facilities, those with a coordinate uncertainty higher than 1 km, duplicates with identical coordinate values, and records unidentified at the species level.

### Species distribution models

We built SDMs to estimate the macroclimatic niche of the species^33^ using the ‘biomod2’ package^73^ in R. We generated models as ensembles of three techniques considered to have a higher prediction accuracy compared with others^74^: Generalized Boosted Models^75^, Random Forests^76^, and Maxent^77^. An ensemble approach using these three techniques was preferred to avoid problems related to the selection of a single modelling algorithm, which can influence results^78^. A total of 21 variables were considered as potential predictors, including the 19 climatic ones from Worldclim 2.0^79^ and two related to vegetation: the Normalized Difference Vegetation Index (NDVI), which was calculated from MODIS (modis.gsfc.nasa.gov) by averaging data from 10-day periods, and the height of the tree canopy^80^. To eliminate multicollinearity between predictors, we calculated the Variance Inflation Factor (VIF) using the package ‘VIF’^81^, discarding variables with a VIF equal to or higher than 10 and keeping the most ecological relevant variables for the study group. Eight variables remained in the final set of predictors for each country (Table 3). Due to computational limitations, variable resolution was proportional to the area of the country of study: 1 km for Costa Rica, 5 km for Mexico and 10 km for the USA. This resolution was maintained for all further analyses. Although using different resolutions may slightly affect the values obtained in the individual representativeness assessment performed for each country, this study does not make comparisons between countries but rather between before and after inclusion of taxonomic groups by country.

**Table 3:** Variables used in the species distribution models for each country The Worldclim variable code (www.worldclim.org) is indicated in parentheses.

	Costa Rica	Mexico	USA
Vegetation	NDVI	NDVI	NDVI
	Canopy height	Canopy height	Canopy height
Temperature	Annual mean (bio 1)	Annual mean (bio 1)	Annual mean (bio 1)
	Seasonality (bio 4)	Mean diurnal range (bio 2)	Mean diurnal range (bio 2)
	Annual range (bio 7)	Isothermality (bio 3)	Seasonality (bio 4)
Precipitation	Annual mean (bio 12)	Annual mean (bio 12)	Annual mean (bio 12)
	Mean wettest month (bio 13)	Mean wettest month (bio 13)	Mean wettest month (bio 13)
	Mean driest month (bio 14)	Mean driest month (bio 14)	Seasonality (bio 15)

We built SDMs using 10,000 background points that were randomly selected from the occurrence records of other species of the same order, a method that has the potential to reduce biases resulting from non-systematic occurrence sampling^82^. For each species, models were fitted with 70% of the occurrence data and validated with the remaining 30%. To increase robustness, we ran 10 replicates for each modelling technique and used the resulting mean in the final model. A final ensemble model was obtained for each species by a weighted averaging of models in each replicate. Weights were calculated from the True Skill Statistic (TSS) of each model, using only those with a TSS > 0.7^83^. Only species whose ensemble model had a TSS > 0.7 were kept, while those whose model had a lower TSS value were discarded. When species were discarded, a new species was analyzed in order to maintain the final number of analyzed species at 1,002 per country (334 per order). The species list, number of occurrences and modelling evaluation parameters can be found as Supplementary Table S1 online.

To limit extrapolations of the model deviating too far from a species’ known area of occurrence, we modified model outputs to restrict them to nearby areas using an exponential decay function^57^. This method is intended to reduce overprediction in areas far from known occurrence areas, simulating the effect of geographic barriers and limitations on species dispersal.

Finally, continuous models were transformed into binary maps (presence/absence) using maximum TSS as a threshold.

### Representativeness assessment

To assess the representativeness of PA networks, a conservation target must be defined for each conservation feature (in our case, species). The conservation target is the proportion of the distribution area of a given conservation feature that should be included in the PA network in order for it to be considered represented. In the case of species, it is considered to be the minimum fraction of the distribution area necessary for the species to thrive^46^. Here, targets were set inversely proportional to the size of the distribution area of each species, i.e. species with narrower distributions had higher targets than widespread species. Building on the approach in^84^, conservation targets ranged between 5% for species with a distribution area larger than 50,000 km^2^ and 80% for those with an area less than 100 km^2^. The lower threshold was set following the B1 criteria (geographic distribution threshold) to declare a species as critically endangered^85^. Conservation targets of species between those thresholds were scaled using a loglinear function^86^.

Once species were assigned a target, we evaluated its achievement in current PAs by comparison with the area of SDM included in the PAs. Species with a distribution inside PAs greater than their target were considered as represented. We classified species that did not achieve their targets in two categories: *underrepresented*, when the area included in the solution was between 50 and 99% of its target, and *neglected*, when the area included in the solution was less than 50% of its target.

### Representation efficiency assessment

We tested the efficiency of PA networks to represent unseen biodiversity from two perspectives: *representation completeness*, to measure the amount of area that needs to be added to an existing PA network to represent all species, and *representation specificity*, to measure the overlap between proposed priority areas that optimize the representation of unseen biodiversity and the existing PA network.

The proposed framework to test efficiency requires contrasting current PA networks against “optimal” areas, which were obtained using the R package ‘prioritizr’ 4.1.1^87^. Prioritizr uses the integer linear programming solver Gurobi Optimizer^88^ to find the combination of sites that maximizes the number of conservation targets achieved while minimizing costs (e.g. the amount of area or the cost of sites).

### Representation completeness

To assess network representation completeness, we executed Prioritizr and forced existing PAs into the solution, which then causes the algorithm to select additional complementary areas as necessary until all species targets are achieved. These solutions can provide information about how much additional area is needed if targets are not met, allowing the efficiency of networks to be measured as a function of the amount of area needed to achieve the desired level of species representation.

### Representation specificity

To assess network representation specificity, a second prioritization was conducted, but without forcing existing PAs into the solution. With this setup, the algorithm identifies the optimal set of sites achieving targets without any prior constraint, which implies that current PAs (or portions of them) might or might not be part of the solution. The overlap between current PAs and resulting areas can then be used to measure how efficient existing PA networks are at representing unseen biodiversity.

## Supplementary Information

Supplementary Information.
